# 中国Ph阴性骨髓增殖性肿瘤患者生活质量及其影响因素分析

**DOI:** 10.3760/cma.j.issn.0253-2727.2021.12.004

**Published:** 2021-12

**Authors:** 梅 宝, 大雨 石, 红霞 石, 晓力 刘, 明辉 段, 俊玲 庄, 新 杜, 玲 秦, 吴函 惠, 蓉 梁, 梅芳 王, 烨 陈, 冬云 李, 威 杨, 古生 唐, 伟华 张, 霞 匡, 伟 苏, 艳秋 韩, 丽梅 陈, 霁虹 许, 卓刚 刘, 健 黄, 春亭 赵, 红艳 佟, 建达 胡, 春燕 陈, 协群 陈, 志坚 肖, 倩 江

**Affiliations:** 1 北京大学人民医院，北京大学血液病研究所，国家血液系统疾病临床医学研究中心，北京 100044 Peking University People's Hospital, Peking University Institute of Hematology, National Clinical Research Center for Hematologic Disease, Beijing 100044, China; 2 南方医科大学南方医院血液科，广州 510080 Nanfang Hospital, Southern Medical University, Guangzhou 510080, China; 3 中国医学科学院北京协和医学院北京协和医院血液内科，北京 100730 Peking Union Medical College Hospital, Chinese Academy of Medical Sciences & Peking Union Medical College, Beijing 100730, China; 4 深圳市第二人民医院，深圳 518035 The Second People's Hospital of Shenzhen, Shenzhen 518035, China; 5 河南科技大学临床医学院/河南科技大学第一附属医院，郑州 471003 The First Affiliated Hospital and College of Clinical Medicine of Henan University of Science and Technology, Zhengzhou 471003, China; 6 首都医科大学宣武医院血液科，北京 100053 Xuanwu Hospital, Capital Medical University, Beijing 100053, China; 7 空军军医大学西京医院血液科，西安 710032 Xi Jing Hospital, The Fourth Military Medical University, Xi An 710032, China; 8 山西医科大学第二医院血液科，太原 030001 Second Hospital of Shanxi Medical University, Taiyuan 030001, China; 9 首都医科大学附属北京安贞医院血液科，北京 100029 Beijing Anzhen Hospital, Capital Medical University, Beijing 100029, China; 10 北京中医药大学东直门医院，北京 100700 Dongzhimen Hospital, Beijing University of Chinese Medicine, Beijing 100700, China; 11 中国医科大学附属盛京医院血液科，沈阳 110020 Shengjing Hospital Affiliated to China Medical University, Shenyang 110020, China; 12 海军军医大学第一附属医院（上海长海医院）血液科，解放军血液病研究所，上海 200433 Department of Hematology, Institute of Hematology, The First Affiliated Hospital of Naval Medical University, Changhai Hospital, Shanghai 200433, China; 13 山西医科大学第一医院血液科，太原 300012 Department of Hematology, First Hospital of Shanxi Medical University, Taiyuan 300012, China; 14 开封市中心医院血液科，开封 475000 Kaifeng Central Hospital, Kaifeng 475000, China; 15 北京中医药大学东方医院，北京 100078 Dongfang Hospital, Beijing University of Chinese Medicine, Beijing 100078, China; 16 内蒙古医科大学附属医院血液内科，呼和浩特 010050 The Affiliated Hospital of Inner Mongolia Medical University, Hohhot 010050, China; 17 西安交通大学第一附属医院血液内科，西安 710061 The First Affiliated Hospital of Xi'an Jiaotong University, Xi'an 710061, China; 18 齐齐哈尔市第一医院血液科，齐齐哈尔 161005 Department of Hematology, Qiqihar First Hospital, Qiqihar 161005, China; 19 浙江大学医学院附属第四医院血液内科，杭州 322000 Department of Hematology, The Fourth Affiliated Hospital of Zhejiang University School of Medicine, Hangzhou 322000, China; 20 青岛大学附属医院血液科，青岛 266003 Department of Hematology, The Affiliated Hospital of Qingdao University, Qingdao 266003, China; 21 浙江大学医学院附属第一医院血液科，杭州 310003 Department of Hematology, The First Affiliated Hospital of College of Medicine, Zhejiang University, Hangzhou 310003, China; 22 福建医科大学附属协和医院血液内科，福州 350001 Department of Hematology, Fujian Medical University Union Hospital, Fuzhou 350001, China; 23 山东大学齐鲁医院血液科，济南 250012 Department of Hematology, Shandong University Qilu Hospital, Jinan 250012, China; 24 西北大学医学院，西安 710069 Institute of Hematology & Affiliated Hospital, Medicine School, Northwestern University, Xi'an 710069, China; 25 中国医学科学院血液病医院（中国医学科学院血液学研究所），实验血液学国家重点实验室，国家血液系统疾病临床医学研究中心，天津 300020 Institute of Hematology and Blood Diseases Hospital, CAMS & PUMC, National Clinical Research Center for Blood Diseases, The State Key Laboratory of Experimental Hematology, Tianjin 300020, China

**Keywords:** 骨髓增殖性肿瘤, 症状负荷, 健康相关生活质量, Myeloproliferative neoplasms, Symptom burden, Health-related quality of life

## Abstract

**目的:**

评估中国Ph阴性骨髓增殖性肿瘤（MPN）患者生活质量及其影响因素。

**方法:**

通过横断面研究，在全国范围内向成年MPN患者发放无记名调查问卷，采用骨髓增殖性肿瘤总症状评估量表（MPN-10）评估症状负荷，健康调查简表（SF-36）和欧洲癌症研究与治疗组织生活质量核心30问卷（EORTC QLQ-C30）量表评估生活质量。

**结果:**

在1405份可评估的调查者问卷中，血小板增多症（ET）、真性红细胞增多症（PV）、骨髓纤维化（MF）受访者分别为645例（45.9％）、297例（21.1％）、463例（33.0％），男性占46.0％（646例），中位年龄56（18～99）岁。ET、PV、MF受访者MPN-10量表评分分别为（13.0±12.7）、（15.0±14.7）、（21.0±16.6）分（*P*<0.001），SF-36量表躯体健康总评分（PCS）分别为（48.0±8.5）、（47.0±9.0）、（42.0±10.0）分（*P*<0.01），精神健康总评分（MCS）分别为（51.0±11.0）、（50.0±10.8）、（49.0±11.1）分（*P*＝0.002）。EORTC QLQ-C30量表中，MF受访者躯体功能、角色功能、情绪功能、认知功能、社会功能和总体健康状况评分最低（*P*值均<0.05），疲劳、疼痛、呼吸困难、食欲丧失、腹泻和经济困难评分最高（*P*值均<0.05）。多因素分析结果显示，三种疾病中，MPN-10评分高（PCS：−0.220～−0.277，*P*值均<0.01；MCS：−0.244～−0.329，*P*值均<0.01）与MPN受访者PCS和MCS低显著相关，年龄增加（−1.923～−4.869，*P*值均<0.05）与PCS低显著相关。此外，共存疾病多、初诊时有症状、脾大、贫血、未知基因突变类型和自付治疗费用高与较低的PCS和（或）MCS相关。年龄≥60岁、城镇户籍、有合并用药、MF受访者采用芦可替尼治疗与较高的MCS相关。在ET、PV和MF受访者中，除食欲丧失和便秘外，MPN-10评分与EORTC QLQ-C30多呈低度相关（ET：|*r*|＝0.193～0.457，*P*值均<0.01；PV：|*r*|＝0.192～0.529，*P*值均<0.01；MF：|*r*|＝ 0.180～0.488，*P*<0.001）。

**结论:**

MPN患者生活质量受损，其中MF患者最差。社会人口学和临床因素显著影响MPN患者的生活质量，其中症状负荷是最重要的影响因素。

## Abstract (en)

**Objective:**

To explore health-related quality of life (HRQoL) and identify its associated variables in Chinese patients with Philadelphia-negative myeloproliferative neoplasms (MPNs).

**Methods:**

In this cross-sectional study, anonymous questionnaires were distributed to adult patients with MPNs to assess symptom burden measured by MPN-10 and HRQoL measured by Medical Outcomes Study 36-Item Short-Form Health Survey (SF-36) and the European Organization for Research and Treatment of Cancer Quality of Life Questionnaire Core 30 (EORTC QLQ-C30).

**Results:**

The data from 1405 respondents with MPNs, including 645 (45.9％) with essential thrombocythemia (ET), 297 (21.1％) with polycythemia vera (PV), and 463 (33.0％) with myelofibrosis (MF), were analyzed. 646 (46.0％) respondents were male. The median age was 56 (range, 18–99) years. The mean MPN-10 scores were 13.0±12.7, 15.0±14.7, and 21.0±16.6 (*P*<0.001), and the physical component summary (PCS) and mental component summary (MCS) scores were 48.0±8.5, 47.0±9.0, and 42.0±10.0 (*P*<0.001) and 51.0±11.0, 50.0±10.8, and 49.0±11.1 (*P*＝0.002) for respondents with ET, PV, and MF, respectively. Respondents with MF reported the lowest score of physical functioning, role functioning, emotional functioning, cognitive functioning, social function, and global health status (all *P*<0.01) and the highest score of fatigue, pain, dyspnea, appetite loss, diarrhea, and financial problems (all *P*<0.05) in EORTC QLQ-C30. Multivariate analyses revealed that higher MPN-10 scores were significantly associated with lower PCS (−0.220 to −0.277, *P*<0.001) and MCS (−0.244 to −0.329, *P*<0.001) scores; increasing age (−1.923 to −4.869; all *P*<0.05), lower PCS score. Additionally, comorbidity (ies), symptom at diagnosis, splenomegaly, anemia, unknown driver gene, and higher annual out-of-pocket cost were significantly associated with lower PCS and/or MCS scores. However, age ≥ 60 years, urban household registration, concomitant medication, and receiving ruxolitinib therapy in respondents with MF were associated with higher MCS scores. Weak correlations were found between MPN-10 score (except the subscale of appetite loss and constipation) and EORTC QLQ-C30 score in majority of subscales in respondents with ET (|*r*| = 0.193–0.457, all *P*<0.001), PV (|*r*| = 0.192–0.529, all *P*<0.01), and MF (|*r*| = 0.180–0.488, all *P*<0.001), respectively.

**Conclusion:**

HRQoL in patients with MPN was significantly reduced, especially in patients with MF. Sociodemographic and clinical variables were significantly associated with the HRQoL in patients with MPNs.

Ph阴性骨髓增殖性肿瘤（MPN）是指分化相对成熟的一系或多系骨髓细胞克隆性增殖所致的一组骨髓肿瘤性疾病，包括原发性血小板增多症（ET）、真性红细胞增多症（PV）和骨髓纤维化（MF）[1]–[2]。MPN患者生存期相对较长，但多受到疲劳、皮肤瘙痒、重度盗汗、发热和体重下降等症状的困扰，因此，患者的健康相关生活质量（HRQoL）越来越受到关注[3]–[5]。HRQoL和症状是患者报告结局（PRO）重要组成部分，是基于生理、心理和社会功能感知的综合健康状况评估，也是癌症等慢性病领域中同一疾病不同治疗结局的评价标准之一[6]–[7]。虽然国外有不少相关研究，但国内大样本研究有限。因此，我们设计了一项横断面研究，以评估中国MPN患者HRQoL并探究其影响因素。

## 病例与方法

一、研究设计

本调研资料获取自一项大型横断面研究。在2017年9月至2021年4月，在全国范围内向MPN患者发放调查问卷，包括通过互联网发放电子问卷及当面发放纸质版问卷。入选标准：①年龄≥18岁；②诊断为ET、PV或MF（包括原发性MF、继发于ET的MF、继发于PV的MF）；③无认知功能障碍。本研究获得北京大学人民医院及各参与单位伦理委员会批准。

二、调研问卷

问卷包括三部分。第一部分包括29个问题：受访者人口学特征（年龄、性别、户籍、婚姻状况、教育程度），共存疾病、合并用药，起病时症状、曾接受的治疗、目前临床症状、疾病病程、血细胞计数、染色体核型、驱动基因突变类型［JAK2、CALR、MPL以及JAK2、CALR、MPL均阴性（三阴性）］、治疗方案，受访者对治疗的满意度，疾病和治疗对生活的影响，自付治疗费用，治疗中的困难，治疗目标，受访者和医生关注的问题等。第二部分为生活质量评估，以健康调查简表（SF-36）和欧洲癌症研究与治疗组织生活质量核心30问卷（EORTC QLQ-C30）量表评估患者填表时的HRQoL。第三部分为症状负荷评估，以骨髓增殖性肿瘤总症状评估量表（MPN-10）评估受访者填表时的症状负荷。在本文中，笔者重点分析受访者HRQoL及其影响因素。

三、症状负荷评估工具

采用MPN-10量表评估受访者症状负荷。MPN-10量表包括10个亚项（疲劳、早饱感、腹部不适、活动力、注意力、夜间盗汗、皮肤瘙痒、骨痛、发热和体重下降），每个项分级为0（无）至10（最严重），总分0～100分。总分越高，表示症状负荷越重[8]。

四、生活质量和症状评估工具

1. SF-36量表：从生理机能、生理职能、躯体疼痛、一般健康状况、精力、社会功能、情感职能和精神健康等8个方面全面概括了受访者的HRQoL，并可概括为躯体健康总评（PCS）和精神健康总评（MCS），每个亚项评分范围为0～100分，评分越高代表健康状况越好[9]。

2. EORTC QLQ-C30量表：一种癌症疾病特异性问卷，用于调查HRQoL，由5个功能量表、3个症状量表、6个单一条目和总体健康状况组成。功能量表包括躯体功能、角色功能、情绪功能、认知功能和社会功能，3个症状量表和6个单一条目包括疲劳、恶心与呕吐、疼痛、呼吸困难、失眠、食欲丧失、便秘、腹泻和经济困难。每个亚项评分范围为0～100分。功能量表评分越高表示功能水平越好，症状量表评分越高表示症状负荷越重。总体健康状况评分越高表示健康状况越好[10]。

五、统计学处理

受访者人口学及疾病特征采用描述性统计分析，组间比较采用Mann-Whitney *U*检验或Kruskal-Wallis检验。单因素分析*P*≤0.2的变量代入多元线性回归模型进行多因素分析。采用皮尔逊相关系数分析MPN-10与EORTC QLQ-C30各个亚项之间的相关性。*P*<0.05被认为差异具有统计学意义。以上统计分析均采用SPSS 22.0软件进行。

## 结果

一、受访者基本特征

2017年9月至2021年4月，共收集1797份调查问卷，受访者来自31个省市自治区和直辖市，剔除受访者年龄未满18岁（27份）、重复填写（102份）、非MPN疾病（69份）和填写不全（194份）的问卷，共有1405份可供评估问卷。1405例可评估受访者的基本特征见表1，其中ET、PV和MF受访者分别为645例（45.9％）、297例（21.1％）和463例（33.0％），男646例（46.0％），中位年龄56（18～99）岁。ET受访者中，女性、年轻、高学历者比例更高；PV受访者，男性、共存疾病、合并用药者比例更高；而MF受访者，高龄、低学历、初诊时有症状、目前有症状、脾大、MPN病程长、自付治疗费用者比例高。ET和PV受访者接受羟基脲治疗患者比例最高、分别为39.5％（255/645）、42.1％（125/297），MF受访者中接受芦可替尼治疗比例为34.1％（158/463）。

**表1:** 1405例Ph阴性骨髓增殖性肿瘤（MPN）患者社会人口学及临床资料

因素	ET（645例）	PV（297例）	MF（463例）	*P*值
男性［例（％）］	248（38.4）	166（55.9）	232（50.1）	<0.001
年龄［岁，*M*（范围）］	52（18~88）	57（20~99）	59（22~84）	<0.001
城镇户籍［例（％）］	428（66.4）	177（59.6）	277（59.8）	0.055
大学及以上学历［例（％）］	252（39.1）	82（27.6）	117（25.3）	<0.001
已婚［例（％）］	568（88.1）	266（89.6）	420（90.7）	0.465
共存疾病数量［例（％）］				<0.001
无	263（40.8）	71（23.9）	164（35.4）	
1种	151（23.4）	84（28.3）	113（24.4）	
2种	132（20.5）	62（20.9）	81（17.5）	
≥3种	99（15.3）	80（26.9）	105（22.7）	
合并用药［例（％）］	555（86.0）	277（93.3）	423（91.4）	0.001
MPN病程［月，*M*（范围）］	15（0~267）	13（0~240）	18（0~336）	0.002
初诊时有症状［例（％）］	353（54.7）	203（68.4）	324（70.0）	<0.001
诊断后接受治疗［例（％）］	576（89.3）	264（88.9）	417（90.1）	0.861
脾大［例（％）］	84（13.0）	95（32.0）	316（68.3）	<0.001
目前有症状［例（％）］	471（73.0）	229（77.1）	411（88.8）	<0.001
MPN-10评分［分，*M*（范围）］	9（0~69）	10（0~80）	17（0~91）	<0.001
WBC［×10^9^/L，*M*（范围）］	8.0（1.0~217.0）	10.0（2.2~169.0）	9.0（1.5~246.0）	<0.001
HGB［g/L，*M*（范围）］	135.0（10.0~329.0）	172.0（20.8~380.0）	113.0（18.0~240.0）	<0.001
PLT［×10^9^/L，*M*（范围）］	619.0（5.8~2127.0）	335.0（4.7~1240.0）	275.0（5.0~2820.0）	<0.001
基因突变［例（％）］				<0.001
JAK2	377（58.4）	228（76.8）	315（68.0）	
CALR	80（12.4）	/	69（14.9）	
MPL	8（1.2）	/	10（2.2）	
三阴^a^	130（20.2）	/	42（9.1）	
未知	50（7.8）	69（23.2）	27（5.8）	
目前治疗［例（％）］				<0.001
阿司匹林单药或未治疗	170（26.4）	77（25.9）	99（21.4）	
羟基脲	255（39.5）	125（42.1）	85（18.4）	
干扰素	194（30.1）	85（28.6）	75（16.2）	
芦可替尼	/	/	158（34.1）	
其他^b^	26（4.0）	10（3.3）	45（9.9）	
自付治疗费用［例（％）］				<0.001
<1万元/年	193（29.9）	85（28.6）	50（10.8）	
1~4万元/年	240（37.2）	124（41.8）	178（38.4）	
>4万元/年	51（7.9）	25（8.4）	170（36.7）	
未知	158（24.5）	63（21.2）	64（13.8）	

注：ET：原发性血小板增多症；PV：真性红细胞增多症；MF：骨髓纤维化。^a^三阴是指JAK2、CALR、MPL均阴性。^b^其他治疗包括化疗、沙利度胺、来那度胺、泼尼松、中药、司坦唑醇、达那唑、促红细胞生成素。/：不适用

二、症状负荷

采用MPN-10量表评估受访者填表时症状负荷。90.3％（1269/1405）的受访者报告至少有1项疾病相关症状，其中，疲劳是最常见的症状之一、发生率为75.0％（1054/1405）。ET、PV和MF受访者MPN-10量表评分分别为（13.0±12.7）、（15.0±14.7）和（21.0±16.6）分（*P*<0.001）。除皮肤瘙痒和发热外，MF受访者在其余症状中评分均最高（*P*值均<0.01）（图1）。

**图1:**
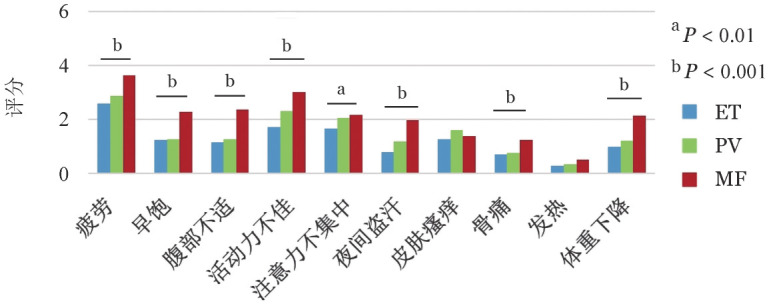
采用骨髓增殖性肿瘤总症状评估量表（MPN-10）评估Ph阴性骨髓增殖性肿瘤患者症状负荷状况 ET：原发性血小板增多症；PV：真性红细胞增多症；MF：骨髓纤维化

三、采用SF-36量表评估生活质量

ET、PV和MF受访者PCS评分分别为（48.0±8.5）、（47.0±9.0）、（42.0±10.0）分（*P*<0.01），MCS评分分别为（51.0±11.0）、（50.0±10.8）、（49.0±11.1）分（*P*＝0.002），MF受访者在每个亚项中评分均最低（图2）。

**图2:**
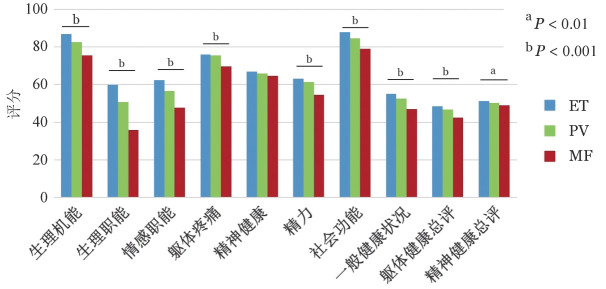
采用健康调查简表（SF-36）评估Ph阴性骨髓增殖性肿瘤患者生活质量 ET：原发性血小板增多症；PV：真性红细胞增多症；MF：骨髓纤维化

将受访者人口学特征（年龄、性别、户籍、婚姻状况和文化程度）、共存疾病、合并用药、初诊时症状、疾病病程、MPN-10评分、血细胞计数、驱动基因突变类型、目前治疗、自付治疗费用纳入单因素分析，分别分析ET、PV和MF受访者PCS和MCS的影响因素。多因素分析结果见表2，三种疾病中，MPN-10评分高（PCS：−0.220～−0.277，*P*值均<0.01；MCS：−0.244～−0.329，*P*值均<0.01）与MPN受访者PCS和MCS低显著相关，年龄增加（−1.923～−4.869，*P*值均<0.05）与PCS低显著相关。此外，共存疾病多、初诊时有症状、脾大、贫血、未知突变类型和自付治疗费用高与较低的PCS和/或MCS评分显著相关。年龄≥60岁、城镇户籍、有合并用药、MF受访者采用芦可替尼治疗与较高的MCS评分显著相关。

**表2:** 中国Ph阴性骨髓增殖性肿瘤患者生活质量影响因素多因素分析结果

因素	ET（645例）				PV（297例）				MF（463例）			
	躯体健康总评		精神健康总评		躯体健康总评		精神健康总评		躯体健康总评		精神健康总评	
	参数估计（SD）	*P*值	参数估计（SD）	*P*值	参数估计（SD）	*P*值	参数估计（SD）	*P*值	参数估计（SD）	*P*值	参数估计（SD）	*P*值
性别（女/男）			−0.624（0.799）	0.435	−1.219（0.913）	0.183	−1.718（1.118）	0.125			−1.731（0.945）	0.068
年龄												
<40岁（参考）												
40~49岁	−1.760（0.929）	0.059	0.434（1.287）	0.736	−1.076（1.805）	0.552			−2.044（1.655）	0.218		
50~59岁	−2.050（0.910）	0.025	1.743（1.225）	0.155	−1.510（1.747）	0.388			−0.894（1.561）	0.567		
60~69岁	−1.923（0.946）	0.042	3.850（1.261）	0.002	−2.348（1.845）	0.204			−2.844（1.559）	0.069		
≥70岁	−3.141（1.086）	0.004	4.267（1.421）	0.003	−4.716（1.949）	0.016			−4.869（1.789）	0.007		
户籍（城镇/农村）			1.762（0.838）	0.036								
婚姻状态												
已婚（参考）												
未婚	−0.757（1.224）	0.537	−0.720（1.692）	0.671	−2.080（2.857）	0.467			4.016（2.184）	0.067		
离异或丧偶	−0.302（1.391）	0.828	3.582（1.919）	0.062	−1.750（1.897）	0.357			2.785（1.806）	0.124		
学历（大学及以上/大学以下）	−0.554（0.572）	0.334							0.929（0.914）	0.310		
共存疾病												
无（参考）												
1种	−0.795（0.758）	0.294			−2.661（1.289）	0.040			−1.409（1.071）	0.189		
2种	−2.132（0.795）	0.008			−2.071（1.409）	0.143			−2.025（1.175）	0.085		
≥3种	−3.259（0.916）	<0.001			−4.856（1.354）	<0.001			−1.125（1.142）	0.325		
合并用药（有/无）							4.557（2.197）	0.039			2.326（1.696）	0.171
MPN病程												
<1年（参考）												
1~4年					−0.332（1.131）	0.769						
>4年					−1.137（1.200）	0.344						
初诊时症状（有/无）	−1.490（0.586）	0.011	−3.407（0.802）	<0.001			−1.378（1.202）	0.253	0.187（0.891）	0.834	−1.091（1.060）	0.304
脾肿大（有/无）	−0.265（0.849）	0.755	−0.642（1.150）	0.577					−1.808（0.919）	0.050	−2.446（1.044）	0.020
WBC												
（4~10）×10^9^/L（参考）											
<4 ×10^9^/L	−1.562（1.219）	0.201							−0.018（1.472）	0.990		
>10 ×10^9^/L	−1.350（0.733）	0.066							−0.345（0.872）	0.693		
HGB												
120~160 g/L（参考）											
<120 g/L	−3.227（0.786）	<0.001					2.583（2.790）	0.355	−2.298（0.920）	0.013	−2.338（1.062）	0.028
>160 g/L	0.457（1.169）	0.696					0.102（1.155）	0.930	1.590（1.422）	0.264	−1.407（1.685）	0.404
PLT												
（100~450）×10^9^/L（参考）											
<100×10^9^/L					0.248（2.826）	0.930			−0.086（1.234）	0.945		
>450×10^9^/L					−1.147（1.062）	0.281			−0.063（1.010）	0.950		
MPN-10评分	−0.277（0.023）	<0.001	−0.318（0.032）	<0.001	−0.220（0.031）	<0.001	−0.329（0.039）	<0.001	−0.253（0.025）	<0.001	−0.244（0.030）	<0.001
基因突变												
JAK2（参考）												
CALR									2.142（1.191）	0.073		
MPL、三阴									−1.630（1.316）	0.216		
未知					3.089（1.225）	0.012			−4.531（1.722）	0.009		
目前治疗												
阿司匹林、未治疗（参考）											
羟基脲	−0.349（0.734）	0.634	−0.165（1.008）	0.870	1.365（1.199）	0.256			0.094（1.330）	0.944	1.827（1.561）	0.243
干扰素	−0.492（0.753）	0.514	−0.855（1.036）	0.410	−0.285（1.343）	0.832			−0.480（1.364）	0.725	0.300（1.610）	0.852
芦可替尼									0.258（1.227）	0.833	3.362（1.466）	0.022
其他	−2.539（1.705）	0.137	3.457（2.350）	0.142	−0.185（3.315）	0.955			−1.113（1.542）	0.471	1.086（1.830）	0.553
自付治疗费用												
<1万/年（参考）												
1~4万/年	−0.581（0.692）	0.402	−1.892（0.964）	0.050	−3.257（1.104）	0.003	−2.141（1.337）	0.110	0.251（1.390）	0.857	−2.350（1.640）	0.152
>4万/年	−0.208（1.169）	0.859	−3.360（1.615）	0.038	−6.516（1.816）	<0.001	−1.651（2.216）	0.457	−1.117（1.553）	0.472	−2.484（1.847）	0.179
未知	−0.484（0.764）	0.527	−2.906（1.059）	0.006	−2.401（1.312）	0.068	−2.436（1.584）	0.125	−0.407（1.659）	0.806	−2.303（1.967）	0.242

注：ET：原发性血小板增多症；PV：真性红细胞增多症；MF：骨髓纤维化；MPN-10量表：骨髓增殖性肿瘤总症状评估量表；其他治疗包括化疗、沙利度胺、来那度胺、泼尼松、中药、司坦唑醇、达那唑、促红细胞生成素等

四、采用EORTC QLQ-C30量表评估评估生活质量

受访者采用EORTC QLQ-C30量表评估HRQoL结果见图3。ET、PV和MF受访者均角色功能和总体健康状况评分最低，经济困难评分最高。ET和PV受访者社会功能和MF受访者认知功能评分最高。MF受访者在躯体功能、角色功能、情绪功能、认知功能、社会功能和总体健康状况评分最低（*P*值均<0.01），疲劳、疼痛、呼吸困难、食欲丧失、腹泻和经济困难评分最高（*P*值均<0.05）。进一步用皮尔逊相关系数分析了MPN-10总分与EORTC QLQ-C30生活质量调查问卷各个亚项相关性。在ET、PV和MF受访者中，除食欲丧失和便秘外，MPN-10评分与EORTC QLQ-C30多呈低度相关（ET：|*r*|＝0.193～0.457，*P*<0.001；PV：|*r*|＝0.192～0.529，*P*值均<0.01；MF：|*r*|＝ 0.180～0.488，*P*值均<0.01）（表3）。

**图3:**
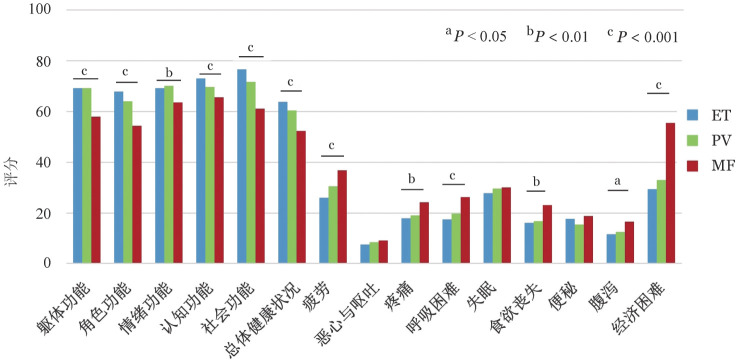
采用欧洲癌症研究与治疗组织生活质量核心30问卷量表（EORTC QLQ-C30）评估Ph阴性骨髓增殖性肿瘤患者生活质量 ET：原发性血小板增多症；PV：真性红细胞增多症；MF：骨髓纤维化

**表3:** MPN-10量表与EORTC QLQ-C30量表亚项之间相关性

评价指标	ET（645例）		PV（297例）		MF（463例）	
	*r*值	*P*值	*r*值	*P*值	*r*值	*P*值
躯体功能	−0.334	<0.001	−0.366	<0.001	−0.312	<0.001
角色功能	−0.387	<0.001	−0.404	<0.001	−0.357	<0.001
情绪功能	−0.429	<0.001	−0.466	<0.001	−0.348	<0.001
认知功能	−0.457	<0.001	−0.529	<0.001	−0.345	<0.001
社会功能	−0.429	<0.001	−0.467	<0.001	−0.391	<0.001
总体健康状况	−0.432	<0.001	−0.443	<0.001	−0.437	<0.001
疲倦	0.428	<0.001	0.393	<0.001	0.484	<0.001
恶心与呕吐	0.303	<0.001	0.222	<0.001	0.331	<0.001
疼痛	0.377	<0.001	0.342	<0.001	0.488	<0.001
呼吸困难	0.374	<0.001	0.192	0.001	0.341	<0.001
失眠	0.267	<0.001	0.256	<0.001	0.302	<0.001
食欲丧失	0.415	0.699	0.254	<0.001	0.422	<0.001
便秘	0.193	<0.001	0.064	0.275	0.180	<0.001
腹泻	0.340	<0.001	0.279	<0.001	0.295	<0.001
经济困难	0.346	<0.001	0.461	<0.001	0.337	<0.001

注：ET：原发性血小板增多症；PV：真性红细胞增多症；MF：骨髓纤维化；MPN-10量表：骨髓增殖性肿瘤总症状评估量表；EORTC QLQ-C30量表：欧洲癌症研究与治疗组织生活质量核心30问卷量表

## 讨论

本研究是一项对中国MPN患者的全国多中心的调研。同文献[11]报道，症状负荷高是严重影响HRQoL的因素，是ET、PV和MF受访者HRQoL受损的共同影响因素。本研究还发现，共存疾病多、初诊时有症状、脾大、贫血、未知突变类型和自付治疗费用高的患者HRQoL差，城镇户籍、有合并用药、MF患者采用芦可替尼治疗与改善HRQoL相关，而年龄增加与较低的PCS和较高的MCS评分相关。

既往研究结果显示MPN患者HRQoL差[11]–[13]，MF患者甚至接近于AML患者[14]。本研究结论与以上研究一致。

本研究中，MPN-10量表和EORTC QLQ-C30量表多呈低度相关，与文献[14]–[15]报道的结果一致。因为MPN-10量表由EORTC QLQ-C30量表演化而来，MPN-10量表主要用于症状负荷评估[16]，而EORTC QLQ-C30量表是评估包括生理、心理、社会、症状负荷、经济在内的HRQoL评估工具[17]，所以两个量表间存在相关性，但MPN-10量表无法全面评估HRQoL。提示，在在临床工作中可以采用MPN-10量表粗略评估患者HRQoL，但不能完全替代HRQoL量表的精准评估。

芦可替尼是一种强效JAK1/JAK2抑制剂，是美国食品药品监督管理局（FDA）批准的首个用于初诊脾大或中高危MF患者治疗的靶向药物[18]。在COMFORT-Ⅰ和COMFORT-Ⅱ研究中，与安慰剂和最佳治疗方案相比，芦可替尼不仅更快、更持久地改善症状，而且伴随改善EORTC QLQ-C30功能量表评分[19]–[20]。本研究也有同样发现，接受芦可替尼治疗的MF患者HRQoL显著高于接受阿司匹林或未治疗的受访者。调研期间，大多数MPN患者通过援助项目接受芦可替尼治疗。即便如此，MPN患者每年自付治疗费用仍高达6万元。众所周知，治疗费用高可对HRQoL产生不利影响[21]–[22]。但本研究中，接受芦可替尼治疗与改善HRQoL相关，说明卢可替尼带来的HRQoL改善抵消了治疗费用高所致的HRQoL受损，显示了芦可替尼治疗对改善生活质量的重要影响。

本研究中，年龄增加与较低的PCS相关。但在ET患者中，年龄增加与较高的MCS评估相关，提示高龄的ET患者精神状态好。可能的原因有：相对于PV和MF患者，ET患者症状负荷轻、病情相对稳定，高龄患者可能已经习惯了慢性疾病的治疗状态。

本研究的主要缺陷：①由于数据分布特征的限制，在进行三组间受访者评分比较时只能采用非参数检验，可能会降低检验效度；②本研究问卷的收集方式使得调研覆盖范围受限，受访者大多来自大中型医院，城镇、高学历人口比例偏高，对农村人口覆盖较少；③受访者对疾病和治疗信息的理解受主观影响较大，缺少客观检查结果；④填表缺失，不够完全。

本研究结果提示，减轻MPN患者症状负荷有望改善其HRQoL，了解MPN患者的HRQoL是提供最佳治疗和评估疗效的重要内容。
